# Future Comorbidities in an Aging Cystic Fibrosis Population

**DOI:** 10.3390/life13061305

**Published:** 2023-05-31

**Authors:** Javier Humberto Ticona, Nicole Lapinel, Janice Wang

**Affiliations:** 1 Department of Medicine, Donald and Barbara Zucker School of Medicine at Hofstra/Northwell, Hempstead, NY 11549, USA; jticona@northwell.edu; 2 Division of Pulmonary, Critical Care and Sleep Medicine, Donald and Barbara Zucker School of Medicine at Hofstra/Northwell, New Hyde Park, NY 11042, USA; nlapinel@northwell.edu

**Keywords:** highly effective modulator therapy (HEMT), screening, dyslipidemia, cardiovascular disease, cystic-fibrosis-related diabetes (CFRD), pulmonary hypertension, obstructive sleep apnea, cystic-fibrosis liver disease (CFLD), osteoporosis, cancer

## Abstract

Cystic fibrosis (CF) is an autosomal recessive disease due to mutations in the cystic fibrosis transmembrane conductance regulator (CFTR) gene. With the advent of highly effective modulator therapy targeting the abnormal CFTR protein, people with CF (PwCF) are living more than 40 years longer than the pre-modulator therapy era. As a result, PwCF are facing new challenges of managing similar comorbidities affecting the average aging population. While CF is notoriously identified as a chronic respiratory disease, the multisystem presence of the CFTR gene can contribute to other organ-related complications acutely, but also heighten the likelihood of chronic conditions not routinely encountered in this cohort. In this overview, we will focus on risk factors and epidemiology for PwCF as they relate to cardiovascular disease, dyslipidemia, CF-related diabetes, pulmonary hypertension, obstructive sleep apnea, CF-liver disease, bone health and malignancy. With increased awareness of diseases affecting a newly aging CF population, a focus on primary and secondary prevention will be imperative to implementing a comprehensive care plan to improve long-term morbidity and mortality.

## 1. Background

**Figure 1:**
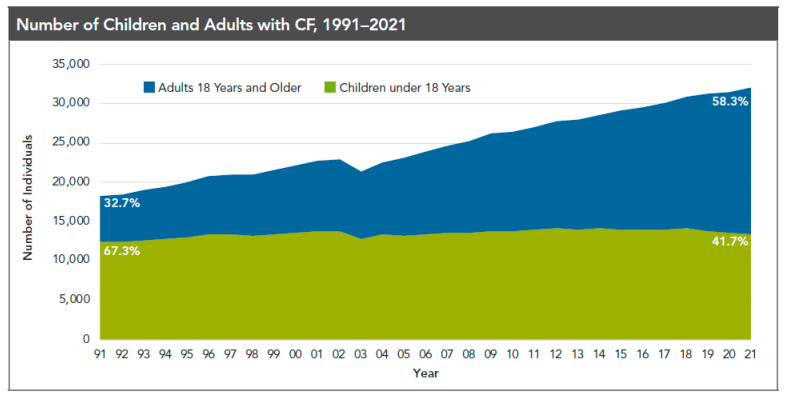
This graph represents cystic fibrosis (CF) patients under care at CF Foundation-accredited care centers in the United States, who consented to have their data entered. The adult cystic fibrosis population is exceeding the pediatric population, indicating the improving life expectancy of people with CF. Reproduced with permission from the Cystic Fibrosis Foundation Patient Registry 2021 Annual Data Report [4].

Cystic fibrosis (CF) is an autosomal recessive genetic disease that was first described in 1938 by Dr. Dorothy H. Anderson at a time when most patients died in childhood [1]. In the 1950s, life expectancy for CF was less than a year, and by the 1970s, a mere 8 years [2]. Worldwide incidence of CF is estimated to be between 1/2000 and 1/6000 with higher rates among people of northern European descent, compared to other races. There are more than 70,000 people with CF (PwCF) worldwide. Approximately 32,000 PwCF live in the United States alone, with more than 1000 cases diagnosed annually [3]. Based on the 2021 U.S. Cystic Fibrosis Foundation (CFF) Patient Registry, 91.4% of registered PwCF are White, 9.8% are Hispanic, 3.5% are African American, and 5.1% are “other” [3,4]. The adult CF population is increasing and exceeding the pediatric population. In the United States, approximately 58% of the CF population are 18 years and older (Figure 1) [4]. CF is caused by mutations in the cystic fibrosis transmembrane conductance regulator gene (CFTR), which is responsible for encoding the CFTR protein, a chloride ion channel located on the epithelial cell surface of multiple organs, including the lungs, sinuses, pancreas, liver, gastrointestinal system, and reproductive system [3,5]. The major mutation responsible for the disease, Delta F508, was first discovered in 1989; however, more than 2000 additional mutations have now been identified [1,5]. Abnormal CFTR protein can lead to an increase in sodium absorption and a relative increase of chloride ion transport into the interstitium. The result is a net increase in sodium chloride absorption and water absorption via an osmotic gradient, ultimately causing impaired mucus hydration and clearance. Although the majority of clinical manifestations in CF are respiratory in nature, the widespread expression of the CFTR channel can lead to complications in other organ systems [5]. Thirty-two percent of adults with CF aged 20–64 can have up to five or more comorbidities [6].

Recent advancements in CF therapies, primarily the CFTR modulators (or highly effective modulator therapy (HEMT)) underscore the impact that personalized medicine has had on redefining treatment paradigms and improving the course of disease. The first CFTR channel modulator approved was ivacaftor in 2012. Lumacaftor then followed in 2015, but was later replaced by tezacaftor, due to side effects, in combination with ivacaftor. The most recent modulator therapy was approved by the United States Food and Drug Administration in 2019. The drug, elexacaftor/tezacaftor/ivacaftor (ETI), is a combination of two protein correctors (elexacaftor and tezacaftor) and one potentiator (ivacaftor) which work to increase CFTR protein activity and mitigate disease progression in patients with at least one F508del mutation. Following the implementation of HEMT, survival for PwCF has improved significantly with the new median predicted age of survival being 53 years for those born with CF today [7]. A person-level microsimulation model based on known clinical outcomes and real-world data on the effectiveness of CFTR modulator therapy was developed to estimate survival and clinical benefits associated with ETI in patients with CF [7]. The median projected survival for patients with CF initiating ETI between ages 12 and 17 years was 82.5 years, a staggering estimate of at least 45 years more than for patients managed with best supportive care. It is imperative that clinicians of all specialties recognize the growing CF patient population and how this aging population will face new medical challenges and morbidities not routinely faced due to their short life expectancies. This chapter will review CF-related diabetes and osteoporosis, which are common comorbidities in CF, as well as those that may benefit from more attention in the future as the patient population grows in age and number: cardiovascular disease, dyslipidemia, pulmonary hypertension, obstructive sleep apnea, CF liver disease, and malignancies. The prevalence of these conditions will likely increase and as such we should be prepared to address them.

## 2. Cardiovascular Disease

The prevalence of cardiovascular disease (CVD) is considered to be low, but may be under-recognized in PwCF given that symptomatic CVD typically presents in older age. The aging adult CF population will begin to face comorbidities commonly associated with increased CVD risk, as seen in the general population, such as obesity, hypertension, cystic-fibrosis-related diabetes (CFRD), and chronic kidney disease [8]. One multicenter cohort of 422 adults with CF and SARS-CoV-19 infection reported a history of ischemic heart disease in about 22.5% of the cohort [9,10]. CFRD likely has the highest prevalence among this population, occurring in about 40% to 50% of PwCF. It is speculated that the traditional diet long recommended for those with CF and failure to gain weight can expose patients to suboptimal food choices that include higher saturated fats and excessive simple carbohydrates intake [11]. Such diets are well known to be associated with development of dyslipidemia and diabetes. Atherosclerosis is inherently a chronic inflammatory process leading to vascular changes such as endothelial damage, deposition of fats, and atherosclerotic plaque formation. CF has been described as a chronic inflammatory disorder, noting that PwCF tend to have higher levels of C-reactive protein (CRP) compared to healthy counterparts [12]. The inflammatory state in CF is multifactorial; not only due to the dysfunctional CFTR, but due to the ensuing chronic respiratory tract infections, as well as local and systemic inflammatory-immune responses. This pro-inflammatory state exhibited in CF may have a cumulative effect on the vasculature, leading to increased oxidative stress, endothelial dysfunction and large arterial stiffness, contributing to early development of hypertension [12,13]. Consequently, heart rate can also be increased in parallel to myocardial work due to increased arterial stiffness. The augmentation index, a composite vascular parameter of arterial stiffness, was found to be elevated in PwCF compared to control groups and increased with aging and/or the presence of CFRD [13,14]. As previously mentioned, the dysfunctional CFTR protein can contribute to chronic inflammation as it stimulates production of interleukin-8 (IL-8), a strong chemotactic agent activating leukocyte infiltration and furthering inflammation [13,14]. CFTR is also found in smooth muscle cells and vascular endothelium; abnormal CFTR activity can disrupt calcium concentration and signaling in vascular smooth muscle cells, resulting in decreased activity of endothelial nitric oxide (NO) synthase and NO bioavailability. Without the vasodilatory, anti-inflammatory and antioxidant properties of NO, there would be an increased risk for progression toward CVD [15]. Interestingly, in mouse models, the restoration of CFTR function reduced total atherosclerosis, improved ischemia-reperfusion injury after cardiac ischemia, and can turn a vulnerable plaque into more stable phenotypes [15]. More awareness is needed for the prevention and treatment of CVD in the aging CF population in order to reduce the future comorbidities that may be related to untreated cardiovascular disease.

## 3. Dyslipidemia

Prevalence of dyslipidemia in PwCF is unclear but likely has been low due to low survival rates and lifetime nutritional challenges. As the life expectancy and longevity of patients with CF increases, dyslipidemia will likely be an additional comorbidity to address [16]. PwCF carry several risk factors for dyslipidemia, including their CF-specific diet, exocrine pancreatic function, CF liver disease, pro-inflammatory state, and corticosteroid exposure. A high-fat, high-calorie diet is a long-established and integral part of CF management, particularly for pancreatic insufficient (PI) patients, alongside the use of pancreatic enzyme replacement therapy (PERT). This diet was first introduced in a Toronto CF clinic in the early 1970s, and was associated with improved nutritional outcomes, thus becoming standard of care through integration into the CF nutritional guidelines [16]. A retrospective cross-sectional review of PwCF performed between 2005 and 2007 showed that pancreatic sufficient (PS) CF patients were more likely to develop dyslipidemia compared to PI-CF patients. These findings translated to triglycerides, low-density lipoprotein (LDL), total cholesterol, and high-density lipoprotein (HDL) levels [16,17]. The significant differences in lipid concentrations seen in this study between PI and PS patients suggest that impaired lipid absorption in PI patients may be a contributing factor. Another study at the University of Minnesota measured the fasting lipid profiles among 192 CF patients and found them to have higher triglyceride and lower cholesterol concentrations when compared to their age-related cohort in the general population. It is hypothesized that hypertriglyceridemia in patients with CF may be related to pro-inflammatory cytokines which promote hyperlipidemia during severe stress by inhibiting lipoprotein lipase activity, thus decreasing triacylglycerol clearance and stimulating hepatic lipogenesis [16,17,18]. There are currently no dyslipidemia screening guidelines in PwCF, but given the findings mentioned, this be serve as a prudent area for development. In the general population, the United States Preventive Services Task Force (USPTSF) strongly recommends that men over the age of 35 and women over the age of 45 get screened at least once for dyslipidemia since there is evidence that people at increased risk for coronary heart disease can be asymptomatic. The question of using lipid-lowering strategies in PwCF needs to be further explored. While dyslipidemia is associated with atherosclerotic CVD in the general population, there has been a paucity of literature on the impact of CVD in PwCF. It is worth noting that atherosclerotic disease has been incidentally found in autopsies of CF patients. General lipid-lowering therapeutics may not be appropriate for all patients with CF given their side effect profiles that include inhibition of fat absorption, body fat reduction, and weight loss [18,19]. Dyslipidemia in CF is heterogenous in presentation, and the underlying molecular mechanisms are not fully understood [20]. For instance, Nowak et al. showed that low HDL was common in both PI- and PS-CF patients, supporting the concept that fat malabsorption alone would not fully explain low HDL levels [20]. Again, chronic low-grade inflammation plays a major role; cytokine TNF-alpha mediated inhibition of hepatic lipoprotein lipase in patients with CF was associated with higher triglyceride levels. The impact of HEMT and associated longer life expectancy warrants further longitudinal studies following lipid profiles and disease development in PwCF to fully understand the risks associated with dyslipidemia in CF [20].

## 4. Cystic Fibrosis Related Diabetes

Cystic fibrosis related diabetes (CFRD) is a common comorbidity among PwCF with an estimated prevalence of 20% in adolescents and up to 40–50% in adults with CF. Survival is significantly impacted by CFRD status, 60% of PwCF with normal glucose tolerance live to age 30, compared to only 25% of those with the disease [21]. The pathophysiology of CFRD is complex, and not fully understood; however, CFRD includes reduced and delayed insulin secretion, insulin resistance, and dysfunction of the entero-insular axis. It is associated with accelerated decline in pulmonary function and worsened nutritional status [21]. Research suggests the CFTR protein plays an important role in insulin and glucagon secretion, as well as protection of β cells from oxidative stress. At present, there is still a lack of consensus on appropriate means to diagnose as well as manage CFRD. As onset of CFRD can occur in early childhood, current recommendations are for screening to begin annually at age 10 with the oral glucose tolerance test (OGTT). HbA1c is not routinely recommended for CFRD diagnosis as it may be falsely normal. More recently, there is an increasing move towards continuous glucose monitoring in select circumstances given its utility in identifying daily glucose fluctuations, finding that its use is more practical and effective [21]. At this time, CFRD is associated more with microvascular (i.e., diabetic retinopathy, nephropathy, neuropathy) than macrovascular complications. Diabetic retinopathy is seen in 5–27% of CF patients with CFRD [21]. It has been found that most CF adults with diabetic retinopathy were diagnosed with CFRD, on average, 12 years prior. It has also been shown that patients with CFRD have increased arterial stiffness. As previously mentioned, arterial stiffness along with hyperlipidemia, hypertension, and external risk factors can contribute considerably to CVD. Case reports have documented CFRD patients suffering from single and multi-vessel cardiac disease [21]. According to the current clinical guidelines, the only recommended treatment for CFRD is insulin [21]. It is hypothesized that older PwCF, particularly with mild CF severity, may develop diabetic manifestations due to a type 2 diabetes-like pathophysiologic pathway (i.e., insulin resistance) rather than from insulin secretory endocrine pancreatic dysfunction as seen more commonly in patients with CFRD. Therefore, with the advent of HEMT, it remains to be seen how CFRD prevalence and management will be affected. CFRD adds another complex condition to CF, already ripe with its distinct complications.

## 5. Pulmonary Hypertension

CF patients develop progressive respiratory disease and/or failure, placing them at increased risk of developing pulmonary hypertension (PH) and subsequent right ventricular dysfunction. The pathophysiology of developing PH in CF is complex and likely multifactorial, including a state of chronic airway and systemic inflammation which can lead to pulmonary vascular remodeling and endothelial dysfunction. Chronic hypoxia is a contributing insult through its vasoconstrictive effects and ensuing effects on pulmonary vascular cell proliferation and pulmonary arteriolar hypertrophy [22]. While PH in CF may be classified as group III, the clinician should also evaluate patients for left ventricular systolic and diastolic dysfunction, particularly in older patients [22,23]. At present, epidemiologic studies using the gold-standard right heart catheterization (RHC) to evaluate for PH in the general CF population are lacking. The predominant research on PH in CF patients have used transthoracic echocardiography (TTE); however, this method can underestimate the true prevalence of PH. A recent transplant study found that 57% of CF patients had PH before lung transplant, but PH did not significantly alter the risk of death after transplant. A study by Hayes et al. found a significant risk of death associated with PH in CF patients, especially in older PwCF [24]. The hazard ratio for mortality risk in CF patients with mild and severe PH were 1.747 and 2.284, respectively (*p* < 0.001). There is a paucity of data investigating PH in the younger CF population, and currently no CF-specific recommendations for PH-directed therapy [24].

Determination for whether PH needs to be screened for at an earlier age is a concept that needs to be further explored in the age of increasing CF survival. It is known that patients with advanced CF lung disease (ACFLD) are at high risk for PH, which is associated with a worse mortality. ACFLD patients should undergo routine screening as these risks play a major role in their candidacy for lung transplantation. In fact, PH is a defining criterion for ACFLD. ACFLD is defined by (1) a forced expiratory volume in one second (FEV1) of <40% predicted when stable; (2) referred for lung transplantation evaluation; or (3) having at least one feature of an intensive care unit admission for respiratory failure, hypercarbia, daytime oxygen requirement at rest, PH, severe functional impairment, and/or a six-minute walk test distance of <400 m [25]. ACFLD care consensus guidelines recommend baseline echocardiography screening and evaluation for hypoxemia. Oxygen supplementation is a mainstay of treatment in order to reduce mPAP, pulmonary vascular resistance and right ventricular dysfunction [25]. While sildenafil, a pulmonary vasodilator, is commonly used in practice and appears to be safe, there remains insufficient evidence for clinical efficacy in patients with ACFLD [25]. Awareness of PH-related signs and symptoms clearly requires particular attention; however, screening is fundamental for early diagnosis and intervention.

## 6. Obstructive Sleep Apnea

Patients with CF commonly exhibit upper airway (UA) inflammation and obstruction. Chronic or acute inflammation is a common cause of UA collapse during sleep, posing a risk for obstructive sleep apnea (OSA) which is associated with intermittent hypoxic events and sleep disruption [26]. Hypoxia can contribute to the development of PH, leading to right ventricular dysfunction or cor pulmonale. Evaluation of nocturnal hypoxia is part of a thorough PH workup to screen and address for risk factors. Sleep-related hypoxemic events have been studied using polysomnography in the younger CF population, including infants and children, with 70% found to have mild to moderate OSA. The most common site of airway obstruction is the oropharyngeal tract and can be due to tonsillar hypertrophy, increased size of uvula, or enlargement of lateral pharyngeal walls. Studies demonstrate a close relationship between OSA and the occurrence of oropharynx structural alteration and chronic rhinosinusitis [26,27]. OSA is strongly associated with CAD, heart failure, hypertension, and cardiac arrhythmias [27]. In adults with CF, OSA can also affect neurobehavioral function as seen with greater levels of fatigue and lower levels of activity and mood. Poor sleep can be a symptoms burden in CF and should be screened routinely with consideration for OSA as a contributing cause. Obesity also predisposes patients to developing OSA, making it another risk to be wary of when caring for PwCF.

## 7. CF Liver Disease

Cystic fibrosis associated liver disease (CFLD) is the third leading cause of mortality in CF patients [28]. It is estimated that the prevalence of CFLD increased from 203.4 to 228.3 per 1000 adult patients during 2008–2013 [28]. Adult-onset CFLD is being increasingly reported, and while the true prevalence of CFLD in adults varies, it may be approaching as high as 40% [28,29,30]. Advanced CFLD with portal hypertension affects about 7% of PwCF [28,29,30]. CFLD is associated with male sex, F508del homozygosity, and previous history of meconium ileus [28]. CFTR dysfunction can give rise to abnormally viscous biliary secretions, ductal obstruction, and subsequent hepatic cirrhosis. It is estimated that up to 40% of patients can develop liver disease throughout their lifetime. CFLD incidence has been shown to increase between 1% and 2% every year from birth up to 25 years of age [28]. A small percentage of these patients will develop cirrhosis within the first decade of their lives. Patients can develop complications from portal hypertension and subsequent variceal bleeding. PwCF and CFLD have more than three times the risk of death compared to those without liver disease [28,30]. Treatment of CFLD with ursodeoxycholic acid has remained controversial, although it is well tolerated and commonly used in practice. There are no current medical therapies available to reverse liver disease in CF, and patients with uncomplicated CF cirrhosis likely do not benefit from liver transplantation. Mendizabal et al. compared the outcomes of 203 patients with CF vs. patients without CF who underwent a liver transplant and concluded that by five years post-transplantation, both children and adults with CF had lower survival rates than non-CF patients [28,31]. The severity of liver disease alone is not sufficient for determining optimal timing for liver transplant, and other factors such as the stage of lung disease must also be strongly considered. The prevalence of CFLD has been increasing in the past few years, and the burden of CFLD has not diminished especially in CF patients in late adolescence and early adulthood. Annual screening with abdominal examinations, radiographic and laboratory testing (e.g., liver function and coagulopathy testing) is recommended in CF patients with CFLD [28,31]. Some hepatic abnormalities include gallbladder changes, hepatic steatosis, and sclerosing cholangitis. Serum markers of liver disease are frequently monitored following the initiation of HEMT. A key area of interest is the long-term effect of HEMT in preventing the development of hepatic fibrosis and advanced CFLD, which will hopefully be elucidated with further observational studies [32]. A diagnosis of liver cirrhosis can be a barrier to lung transplantation; thus, it is important to understand CFLD in order to develop new strategies to prevent progressive liver damage.

## 8. Bone Health

CF bone disease (CFBD), primarily due to osteoporosis, is a well-recognized morbidity in CF, the prevalence for which can be guaranteed to increase among the aging CF population. Fracture risks have been estimated to be twice as high in PwCF compared to the general population. Poor bone health contributes to increased prevalence of kyphosis in PwCF and reduced lung function, and negatively affects tolerability to perform effective airway clearance techniques [33]. CFBD affects less than 5% of children with CF, 20% of adolescents with CF, and up to 65% of patients older than 45 years [34]. Bone mineral density (BMD) in the pediatric CF population age is half that of the non-CF population [34]. In young adults with CF, average BMD of the spine and distal radius is also reduced [34]. These findings have not changed over a 15-year period since 1995, despite improvements in lung function and with diagnosis and treatment for vitamin D deficiencies [35] This observation demonstrates that the underlying predisposition for CFBD is multifactorial and includes not only vitamin D deficiency and a chronic inflammatory state from CF lung disease, but may also be attributed to other established causes such as pancreatic insufficiency, vitamin K deficiency, systemic corticosteroid use, CFRD, hypogonadism, malnutrition, and physical inactivity [35]. Intermittent episodes of pulmonary exacerbations (Pex) play a role by driving inflammatory cytokine levels (e.g., tumor necrosis factor- α) which potentially leads to increased levels of osteoclast precursors and increased osteoclast activity, bone resorption, and decreased bone formation [36,37]. With antibiotic treatment for Pex, osteoclast activity is reduced back to baseline; however, cumulative bone loss will have occurred over time with every Pex.

The CFF consensus statement recommends BMD screening with dual energy X-ray absorptiometry (DXA) as the gold standard for all adults 18 years and older with CF and for children at least 8 years old with any of the following clinical criteria: (1) ideal body weight is less than 90% of the goal; (2) forced expiratory volume in 1 s (FEV1) is less than 50% predicted; (3) glucocorticoid use of greater than or equal to 5 mg/day for at least 90 days per year; (4) delayed puberty; and/or (5) a history of fractures [38]. At 20 years of age and older, BMD is measured at the lumbar spine and proximal hip (see Table 1 for CFF guidelines for bone health screening). Preventative care through screening, early diagnosis, and treatment are an essential part of CF care. Nutritional management is a major part of bone health, and registered dieticians within CF care centers and clinicians should be attentive to routine monitoring. Treatment includes bisphosphonates such as alendronate, risedronate, and ibandronate, which act by inducing osteoclast apoptosis, thereby inhibiting bone resorption [38]. Another available treatment is denosumab, a monoclonal antibody against receptor activator of nuclear factor ĸβ ligand (RANKL), that inhibits osteoclast formation and reduces bone resorption.

## 9. Cancer

Prior to therapeutic advancements with HEMT, the prevalence of cancer in CF was rare as it was primarily a pediatric disease due to short life expectancy. Between 1971 and 1986, a study in the United Kingdom identified 7 children with CF out of 20,071 children registered with childhood cancer [40]. With improved life expectancy and more than half of the CF population now living to an adult age, cancer incidence will likely increase with age. The U.S. CFF Patient Registry data from 1990 to 2009 revealed an increased prevalence of digestive tract cancer, testicular cancer, and lymphoid leukemia [41]. There are several potential risks for cancer in PwCF. Repeated exposure to radiation from X-rays and CT scanning over the lifetime of a person with CF is concerning for the development of acute lymphoblastic leukemia and thyroid cancer [41]. Another mechanism for increased cancer risk lies in the evidence that the CFTR gene serves as a tumor suppressor gene in the intestinal tract in humans and animal models [42,43]. Reduced CFTR expression and carrier status for CFTR mutation, even in people without a clinical diagnosis of CF, are recognized as risk factors for colorectal cancer (CRC), cancers of the gallbladder and biliary tract, thyroid cancer, and unspecified NHL [42,43,44].

Reported digestive tract cancers include those of the esophago-gastric junction, biliary tract, small bowel, and colon. The risk of CRC in adults with CF is 5–10 times greater than the general population and is associated with carrier status for the more severe CFTR mutations, (classes I, II, and III) compared to the milder classes (IV and V) which have residual CFTR function. CF comorbidities and the pathophysiology itself also contribute risk to digestive tract cancers. For example, dysfunctional CFTR activity at the intestinal epithelia level can lead to dehydration, promote bacterial overgrowth, which can disrupt the gut microbiome and epithelial turnover, contributing to cancer risk [41,45,46]. Common CF comorbidities of diabetes, gastroesophageal reflux, inflammatory bowel disease, vitamin D deficiency, and high-fat diets are recognized risk factors for digestive tract cancers.

Through a multidisciplinary task force, the CFF published CRC screening recommendations specific to PwCF, recommending colonoscopy as the screening examination in PwCF beginning at age 40, with re-screening every 5 years. Earlier re-screening by 5 years as opposed to 10 years in the general population is based on the high rate of adenomatous polyps and advanced adenomas within 5 years [46,47]. Bowel preparation for colonoscopy requires a more intensive regimen, consisting of multiple small-volume washes over one larger volume regimen to fully optimize colonoscopic visualization [45,47]. Procedures other than colonoscopy are not recommended at this time; flexible sigmoidoscopy is limited to the descending colon, and computed tomography (CT) colonography does not provide a means for biopsy. Currently, there are not enough data to recommend stool-based tests. Positive screening with adenomatous polyps requires surveillance colonoscopy in 3 years or sooner if indicated. CF recipients of solid organ transplants who are 30 years of age or older and have fully recovered from their transplant should have CRC screening within 2 years of transplant unless they had a negative colonoscopy within the past 5 years. Rescreening for transplant recipients is every 5 years.

PwCF who are post-lung transplant recipients are at a 10-fold higher overall cancer risk compared to the general population, with the most common cancer among CF patients being non-Hodgkin lymphoma (NHL), followed by CRC [48]. Post-transplant lymphoproliferative disease (PTLD) consists mainly of abnormal B-cell proliferation occurring within one year of transplant [49]. Based on the International Society for Health and Lung Transplantation Registry, 17% of a cohort of 30,598 adult recipients of lung transplants between 1999 and 2011 had CF; PTLD developed in 2% of CF recipients compared to 1% for non-CF recipients in this cohort [50]. Recipient seronegativity for Epstein–Barr virus (EBV) and cytomegalovirus (CMV), and EBV and CMV mismatch were found to be of higher prevalence among CF compared to non-CF patients in the cohort. Awareness of PTLD is crucial as clinical presentation can be extremely variable. Mainstay treatments include antiviral medications and immunoglobulin therapy, reduction of immunosuppression, surgical resection and irradiation therapy, and chemotherapy [50].

Since HEMT enhances CFTR activity, it is hypothesized that long-term use may reduce cancer risk; however, this remains to be seen as we follow registry data and future comorbidities. Certain malignancies are known to be of higher prevalence in the CF compared to the general population, and therefore need special consideration. Clinicians should be aware of important cancer screenings that are both unique to PwCF, but also the standard of care in the general population.

## 10. Conclusions

The advent of HEMT over the last decade has changed the course of treatment, improving quality of life through exacerbation prevention, but also improving life expectancy by decades for PwCF. Until a cure for CF is found, morbidity directly related to CF remains significant. The CF population has always needed to contend with unique risk factors affecting morbidity and mortality, but as a population that is beginning to experience increased longevity, with this will come the need for new screening guidelines focusing on preventative care. The general population not afflicted with CF has important guideline-based screening measures for diabetes, dyslipidemia, cardiovascular health, bone health and malignancy. All of these same considerations must now be applied to the aging CF population, but with consideration for the pathophysiology which raises their inherent likelihood for adverse outcomes. More research is necessary to follow PwCF longitudinally to devise proper population-specific screening guidelines. In the interim, CF care teams must remain vigilant in the care of PwCF according to accepted guidelines, which will likely evolve with future incorporation of age-related comorbidities.

## Figures and Tables

**Table 1:** Cystic Fibrosis Foundation guideline recommendations for bone health monitoring in people with CF [39]. CFRD = cystic fibrosis related diabetes; Pex = pulmonary exacerbation; BMD = bone mineral density.

BMD T/Z Score	Interpretation	Management	Follow-Up DXA Screening
≥1.0	Normal	● Monitor ● Maintain nutritional health	Repeat screening every 5 years
Between −1.0 and −2.0	Osteopenia	● Address calcium, vitamin D insufficiency ● Minimize corticosteroid use ● Improve glycemic control in CFRD ● Optimize respiratory health to reduce Pex ● Consider bisphosphonate therapy in patients with history of fragility fractures, BMD loss of > 3–5%, and/or are awaiting transplant	Repeat every 2 to 4 years
≤−2.0	Osteoporosis	● Start treatment (e.g., bisphosphonate, denosumab)	Annually
